# Socioeconomic and Racial Inequities in Breast Cancer Screening During the COVID-19 Pandemic in Washington State

**DOI:** 10.1001/jamanetworkopen.2021.10946

**Published:** 2021-05-24

**Authors:** Ofer Amram, Jeanne Robison, Solmaz Amiri, Bethann Pflugeisen, John Roll, Pablo Monsivais

**Affiliations:** 1 Department of Nutrition and Exercise Physiology, Elson S. Floyd College of Medicine, Washington State University, Spokane; 2 Paul G. Allen School for Global Animal Health, Washington State University, Pullman; 3 Multicare Institute for Research & Innovation, Tacoma, Washington; 4 Multicare Deaconess Cancer & Blood Specialty Centers, Spokane, Washington

## Abstract

This cohort study investigates the rates of breast cancer screening before and during the COVID-19 pandemic among women in Washington State.

## Introduction

The COVID-19 pandemic has disrupted preventive care, including cancer screening. Studies from the United States and Europe have shown that cancer screening dropped dramatically during the pandemic,^1,2^ with breast cancer screening and diagnostic mammograms falling by 58% and 38%, respectively.^1,2^ A United Kingdom modeling study estimated that delayed and missed screenings would likely increase breast cancer deaths, a leading cancer among women, by 7.9% to 9.6%.^2,3^ The adverse impact of COVID-19 on screening may differ among sociodemographic groups, given the disproportionate impact the pandemic has had on underserved racial and ethnic groups and other vulnerable population groups.^4^ In this report, we used clinical data to examine differences in breast cancer screenings before and during the COVID-19 pandemic overall and among sociodemographic population groups.

## Methods

### Data

Data included completed screening mammograms within a large statewide nonprofit community health care system in Washington State between April 1, 2018, and December 31, 2020. This health care system included more than 230 primary care, specialty care, and urgent care clinics, and 8 hospitals across Washington State. The MultiCare institutional review board approved this study protocol and granted waivers of individual consent based on removal of individually identifying data. This study followed the Strengthening the Reporting of Observational Studies in Epidemiology (STROBE) reporting guideline.

### Measures and Statistical Analysis

Sociodemographic data included patients’ race and ethnicity, insurance, and zip code of residence. Rural-urban commuting area codes differentiated between urban vs rural residence. Inclusion criteria included women who had at least 1 screening mammogram within the health system in 2018 or 2019. Frequency analysis and χ^2^ tests were performed using a significance level of *P* < .05 to test for differences in screening in 2019 and 2020. Testing was 2-sided. Statistical analysis was performed using R statistical software version 4.03 (R Project for Statistical Computing).

## Results

Among the 55 678 screenings in April to December 2019, 45 572 patients were non-Hispanic White (81.8%), 54 620 patients lived in urban areas (98.1%), and 22 761 patients were commercially insured (40.9%); the mean (SD) age was 62.0 (11.3) years. From 2019 to the same period in 2020, there was a 49% decrease in screenings (55 678 screenings in 2019 vs 27 522 screenings in 2020), with some differences apparent in the demographic characteristics between the 2 years (Table). We observed greater and significant reductions in the number of screenings from 2019 to 2020 for women who were Hispanic (1727 vs 619; −64.2%), American Indian/Alaska Native (215 vs 84; −60.9%), mixed race (1892 vs 828; −56.2%), Native Hawaiian or Pacific Islander (365 vs 166; −54.5%), Asian (2779 vs 1265; −54.5%), and Black (2320 vs 1069; −53.9%) compared with women who were White (45 572 vs 23 163; −49.2%) (Figure). Women living in rural areas experienced greater reduction in screenings compared with their urban counterparts. In terms of insurance, women who self-paid for treatment and who were insured by Medicaid experienced the largest reduction in screening, whereas those with commercial insurance or Medicare showed smaller reductions (Table).

**Table.** Characteristics of Patients Undergoing Screening Mammograms During April to December in 2019 and 2020

Variable	Screenings, No. (%)	
	2019 (n = 55 678)	2020 (n = 27 522)
Age, mean (SD), y	62.0 (11.3)	62.8 (11.0)
<50	9020 (16.2)	3748 (13.9)
50-64	22 343 (40.1)	10 871 (39.5)
≥65	24 315 (43.7)	12 903 (46.9)
Racial category		
Non-Hispanic White	45 572 (81.8)	23 163 (84.2)
Black	2320 (4.2)	1069 (3.9)
NHPI	365 (0.7)	166 (0.6)
AIAN	215 (0.4)	84 (0.3)
Hispanic of any race	1727 (3.1)	619 (2.2)
Asian	2779 (5.0)	1265 (4.6)
Mixed race	1892 (3.4)	828 (3)
Geography		
Urban^a^	54 620 (98.1)	27 089 (98.4)
Rural^b^	1046 (1.9)	431 (1.6)
Insurance		
Commercial^c^	22 761 (40.9)	11 378 (41.3)
Government^d^	894 (1.6)	421 (1.5)
Medicaid	2503 (4.5)	931 (3.4)
Medicare	19 847 (35.6)	10 614 (38.6)
Self-pay^e^	483 (0.9)	142 (0.5)

Abbreviations: AIAN, American Indian/Alaska Native; NHPI, Native Hawaiian or Pacific Islander.

^a^ Urban based on Rural-Urban Commuting Area classification 1-3.

^b^ Rural based on Rural-Urban Commuting Area classification 4-10.

^c^ Commercial insurance was administered by nongovernmental entity.

^d^ Government insurance was administered by a governmental entity.

^e^ Self-pay patients paid out-of-pocket and were largely uninsured and had lower socioeconomic status.

**Figure.**
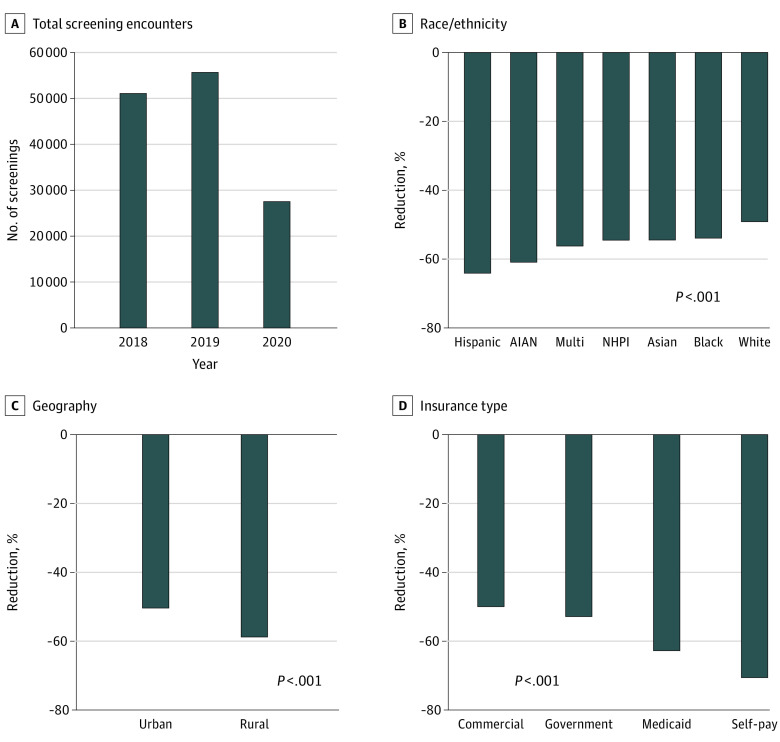
Reductions in Screening Mammograms Reductions in screening mammograms for April-December 2018-2020 (A) and changes between 2019 and 2020 for patients by race (B), by rural or urban residence (C), and by type of insurance coverage (D). *P* values from χ^2^ tests comparing 2019 and 2020 values. Urban based on Rural-Urban Commuting Area classification 1-3; rural based on Rural-Urban Commuting Area classification 4-10. Commercial insurance was administered by non-governmental entity; government insurance was administered by a governmental entity; self-pay patients paid out-of-pocket and were largely uninsured and had lower socioeconomic status. AIAN denotes American Indian/Alaska Native; multi, mixed race; NHPI, Native Hawaiian or Pacific Islander.

## Discussion

This study found a substantial overall decline in breast cancer screening in women living in Washington State during the COVID-19 pandemic, as well as inequities in this decline. This study has several limitations. First, the analyses were of aggregate data; we did not link individual records across years. Second, the demographic characteristics of this sample are slightly less diverse and more affluent than Washington State. Third, data reflect patient interactions with a single health care system; we weren’t able to link these interactions to an underlying population base. However, the substantial drop in screenings in 2020 was not likely to be explained by a drop in underlying population base or eligibility; nor was it likely the result of a shift to different health care networks, given that this clinical network is one of the largest health care systems in Washington State.

The larger decline in screening among women from underserved racial/ethnic groups and lower socioeconomic status might be explained by several factors. Increasing unemployment during the pandemic shutdown among those already living in poverty may have further reduced access to health insurance, while school closures led to competing demands at home.^5^ In addition, limited access to health and screening services among rural women may have increased during the pandemic.^6^ To address the decline in breast cancer screening during the pandemic, there is a need to address barriers to screening, especially for higher-risk women. Our findings suggest another inequity in the COVID pandemic due to greater reduction in utilization of cancer screening services for women with lower socioeconomic status, who are in underserved racial/ethnic groups, and live in rural communities.

## References

[1] Song H, Bergman A, Chen AT, . Disruptions in preventive care: mammograms during the COVID-19 pandemic. Health Serv Res. 2021;56(1):95-101. doi:10.1111/1475-6773.13596 33146429 PMC7839639

[2] Maringe C, Spicer J, Morris M, . The impact of the COVID-19 pandemic on cancer deaths due to delays in diagnosis in England, UK: a national, population-based, modelling study. Lancet Oncol. 2020;21(8):1023-1034. doi:10.1016/S1470-2045(20)30388-0 32702310 PMC7417808

[3] American Cancer Society. Breast cancer facts & figures 2019-2020. Published 2019. Accessed May 3, 2021. https://www.cancer.org/content/dam/cancer-org/research/cancer-facts-and-statistics/breast-cancer-facts-and-figures/breast-cancer-facts-and-figures-2019-2020.pdf

[4] Centers for Disease Control and Prevention. COVID-19 racial and ethnic health disparities. Published February 11, 2020. Accessed March 21, 2021. https://www.cdc.gov/coronavirus/2019-ncov/community/health-equity/racial-ethnic-disparities/index.html

[5] Gezici A, Ozay O. How race and gender shape COVID-19 unemployment probability. Social Science Research Network. Published August 17, 2020. Accessed April 12, 2021. doi:10.2139/ssrn.3675022

[6] Jewett PI, Gangnon RE, Elkin E, . Geographic access to mammography facilities and frequency of mammography screening. Ann Epidemiol. 2018;28(2):65-71.e2. doi:10.1016/j.annepidem.2017.11.012 29439783 PMC5819606

